# Structure of plant photosystem I in a native assembly state defines PsaF as a regulatory checkpoint

**DOI:** 10.1038/s41477-024-01699-8

**Published:** 2024-05-30

**Authors:** Andreas Naschberger, Mariia Fadeeva, Daniel Klaiman, Anna Borovikova-Sheinker, Ido Caspy, Nathan Nelson, Alexey Amunts

**Affiliations:** 1 Science for Life Laboratory, Department of Biochemistry and Biophysics, Stockholm University, Solna, Sweden; 2 The George S. Wise Faculty of Life Sciences, Department of Biochemistry and Molecular Biology, Tel Aviv University, Tel Aviv, Israel; 3 Present Address: Westlake University, Hangzhou, China

**Keywords:** Cryoelectron microscopy, Photosystem I

## Abstract

Plant photosystem I (PSI) consists of at least 13 nuclear-encoded and 4 chloroplast-encoded subunits that together act as a sunlight-driven oxidoreductase. Here we report the structure of a PSI assembly intermediate that we isolated from greening oat seedlings. The assembly intermediate shows an absence of at least eight subunits, including PsaF and LHCI, and lacks photoreduction activity. The data show that PsaF is a regulatory checkpoint that promotes the assembly of LHCI, effectively coupling biogenesis to function.

## Abstract (web summary)

This study reports the structure of a photosystem I assembly intermediate isolated from greening oat seedlings. It defines PsaF as a regulatory checkpoint promoting the association of LHCI that couples biogenesis to function.

## Main

Photosystem I (PSI) is a large protein–pigment complex embedded in photosynthetic membranes of chloroplast and cyanobacteria. It primarily functions as a transmembrane electron conductor from the luminal reduced carrier plastocyanin (Pc) to the stromal oxidized acceptor ferredoxin, providing the reducing power needed for carbon fixation. Chloroplast PSI shares a common ancestor with its cyanobacterial counterpart and has additional subunits and light-harvesting proteins that can be remodelled to regulate dimerization^1,2^. In plants, the subunits PsaA–PsaC, PsaI and PsaJ are encoded in the chloroplast genome, whereas at least 13 tightly bound subunits are imported from the cytoplasm. Subunit PsaF is unique with respect to its location in the complex and the assembly path. It is stabilized by a single transmembrane subunit PsaJ in a way that orients the amino-terminal (N-terminal) domain towards the lumen, where it contributes to the docking site for Pc^3–5^. The assembly path of PsaF involves import into the thylakoid lumen^6^. In this way, a colocalization of the positively charged N-terminal region with the Pc is achieved, thus supporting the functional recognition of Psaf and Pc for electron transfer that is based on electrostatic interactions^2,7,8^.

The complex architecture of PSI and the requirement to coordinate two genetic systems and distinct assembly paths generate additional complexity for the biogenesis of PSI. A stepwise process has been suggested^9^, and biochemical studies in *Chlamydomonas reinhardtii* showed that *trans*-acting factors contribute to biogenesis^10^. In particular, chloroplast-encoded Ycf3 and Ycf4 form modules that mediate PSI assembly. Ycf3–Y3IP1 mainly facilitates the assembly of PsaA–PsaB, leading to the reaction centre subcomplex, and oligomeric Ycf4 facilitates the integration of peripheral PSI subunits. However, despite the central role of PSI in the light reaction of photosynthesis, in plants the data are limited to biochemical characterizations of subcomplexes in young leaves of *Nicotiana tabacum*^11^, and there are no structural data on any of the assembly steps of PSI.

**Fig. 1:**
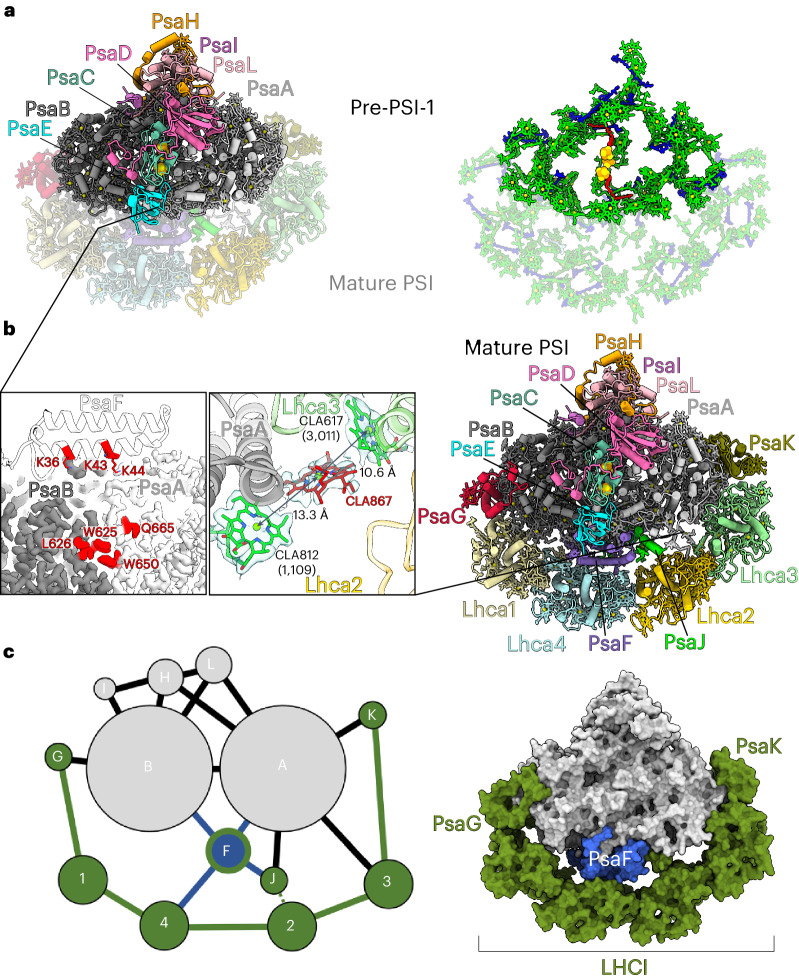
Structural characterization of the PSI assembly intermediate. **a**, Model of pre-PSI-1 with missing PsaF and LHCI. Left: complete model coloured by individual subunits. Right: model of cofactors. The mature PSI model is shown as a semitransparent background layer. **b**, Left: close-up view of pre-PSI-1 shows the missing PsaF in white cartoon and the PsaA–PsaB region with Pc-binding residues in red. Right: mature PSI with newly identified chlorophyll CLA867 in red cartoon and previously modelled CLA617 and CLA812 in green cartoon; the corresponding cryo-EM density is shown in the close-up view. Mg–Mg distances between chlorophylls are indicated, suggesting a potential excitation energy pathway. **c**, Left: schematic of pre-PSI-1 and mature PSI protein–protein interactions of membrane subunits. The node size corresponds to relative molecular mass of protein subunits, and the connector length corresponds to the solvent accessible interface area buried between the subunits, calculated with PDBePISA v.1.52 (ref. ^12^). Interactions of PsaF are shown in blue, and interactions between late assembly subunits are shown in green. PsaJ and Lhca2 are connected via cofactors, shown as a dashed line. Right: surface representation of the model. Only transmembrane subunits are shown: pre-PSI-1 subunits (grey), PsaF (blue) and late assembly subunits of mature PSI (green).

**Extended Data Fig. 1:**
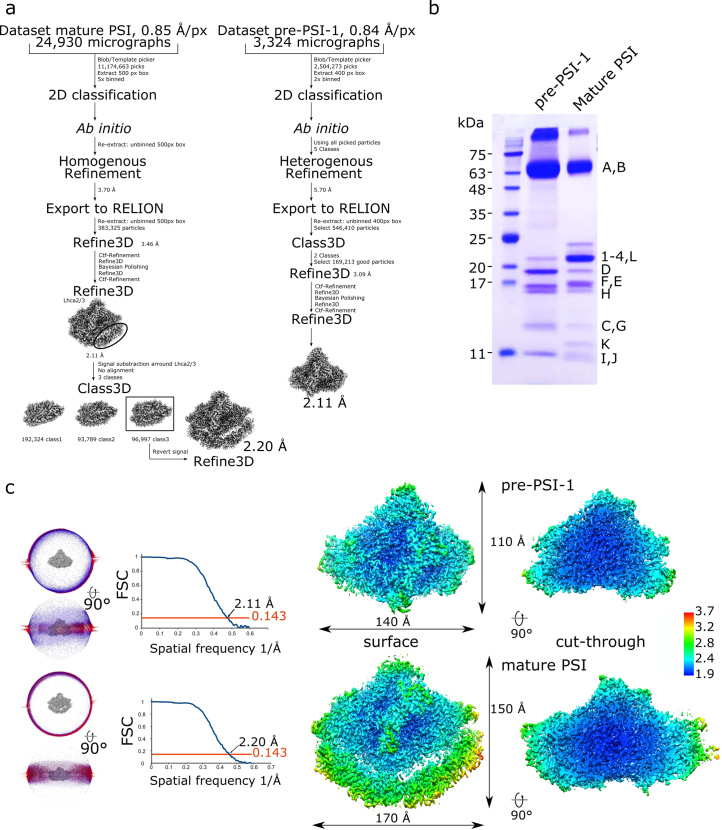
Data processing, Fourier Shell Correlations (FSCs), angular distributions. **a**, Data processing scheme for mature PSI on the left and pre-PSI-1 on the right. **b**, Coomassie stained SDS-PAGE of pre-PSI-1 and mature PSI. The experiments were repeated independently with similar results three times. **c**, Angular distribution and FSC for resolution estimation for pre-PSI-1 (top) and of mature PSI (bottom). Local resolution of 3D reconstructed map of pre-PSI-1 and mature PSI.

To identify a stable PSI assembly intermediate, we took advantage of the etioplast-to-chloroplast transition system. We grew *Avena sativa* seedlings in darkness to accumulate photosynthetically inactive organelles, and then initiated greening by irradiating the seedlings as they gained enough etiolated biomass. At 10 h of irradiation, we harvested the plants, isolated thylakoids and solubilized photosynthetic complexes. The material that migrated as a green band was detected on a sucrose gradient, eluted and subjected to cryogenic electron microscopy (cryo-EM) analysis. We collected 3,324 images and generated a reconstruction at 2.1 Å resolution (Extended Data Fig. 1 and Table 1). The map dimensions of 140 Å × 110 Å compared with a typical size of 170 Å × 150 Å for a mature PSI suggested a different protein composition with missing subunits (Extended Data Fig. 1b,c). The well-defined density allowed us to build an atomic model that corresponds to a subcomplex consisting of eight subunits, PsaA, PsaB, PsaC, PsaD, PsaE, PsaH, PsaI and PsaL, which we name pre-PSI-1 (Fig. 1a). The pre-PSI-1 assembly intermediate lacks the antenna LHCI and all the subunits related to its association: PsaF, PsaG, PsaJ, PsaK and PsaN.

**Table 1:** Cryo-EM data collection, refinement and validation statistics of PSI and pre-PSI-1 assembly intermediate of *A*. *sativa*

	PSI (EMDB-15969; PDB: 8BCV)	pre-PSI-1 (EMDB-15970; PDB: 8BCW)
**Data collection and processing**		
Magnification	105,000	105,000
Voltage (kV)	300	300
Electron exposure (e^−^ Å^−2^)	45	51.35
Defocus range (μm)	−0.5 to −1.9	−0.5 to −1.9
Pixel size (Å)	0.85	0.84
Initial particle images (*n*)	24,930	3,324
Symmetry imposed	*C*_1_	*C*_1_
Final particle images (*n*)	96,997	169,213
Map resolution (Å)	2.2	2.11
**Refinement**		
Initial model used (PDB code)	6YAC	6YAC
Map sharpening *B* factor (Å^2^)	−17.41	−21.56
Model composition		
Non-hydrogen atoms	37,485	22,313
Protein residues	3,258	2,029
Ligands	224	114
Waters	703	517
* B* factors (Å^2^) (minimum/maximum/mean)		
Protein	12.15/68.29/33.23	8.91/61.68/28.79
Ligand	20.51/82.86/38.02	8.86/70.11/29.53
Waters	13.12/42.46/27.46	11.46/43.71/26.66
r.m.s. deviations		
Bond lengths (Å)	0.009	0.008
Bond angles (°)	1.053	1.034
Validation		
MolProbity score	1.23	1.09
Clashscore	4.57	2.7
Poor rotamers (%)	0.76	0.6
Ramachandran plot (%)		
Favoured	98.3	97.86
Allowed	1.67	2.14
Disallowed	0.03	0

r.m.s., root mean square.

**Fig. 2:**
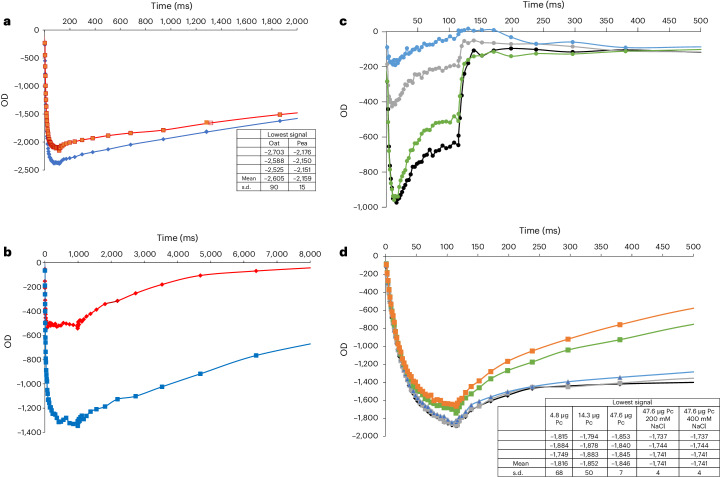
Biochemical and kinetic characterization of the PSI assembly intermediate. **a**, Light-induced P700 oxidation in mature PSI preparations from *P. sativum* (red) and *A. sativa* (blue). Results of three independent measurements of light-induced P700 photo-oxidation in respective experiments are shown in the table. **b**, Chlorophyll/P700 ratio of the *A. sativa* pre-PSI-1 (red) that contains 88 chlorophylls compared with mature PSI from *P. sativum* with 154 chlorophylls (blue). **c**, Light-induced P700 photo-oxidation and P700^+^ reduction of mature PSI from *A. sativa* with different Pc concentrations: 4.8 µg (black), 14.3 µg (grey), 47.6 µg (blue) and 47.6 µg (green) of Pc + 200 mM NaCl. **d**, Light-induced P700 photo-oxidation and P700^+^ reduction of pre-PSI-1 with different Pc concentrations: 4.8 µg (black), 14.3 µg (grey), 47.6 µg (blue), and 47.6 µg (green) of Pc + 200 mM NaCl, and 47.6 µg of PC + 400 mM NaCl (orange). Results of three independent measurements of light-induced P700 photo-oxidation in respective experiments are shown in the table. OD, optical density.

**Extended Data Fig. 2:**
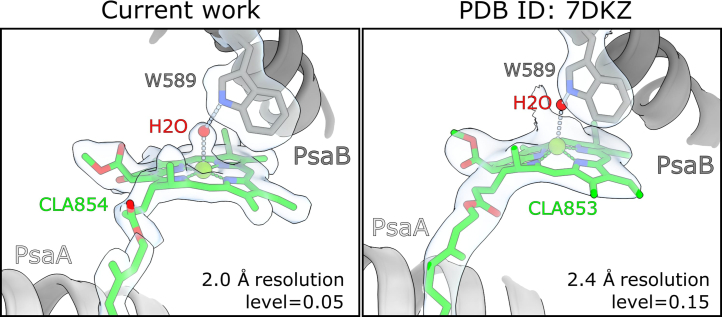
Cryo-EM map quality. Left, the current density around CLA854 is shown in iso-surface representation at a local resolution of 2.0 Å. A water molecule involved in the chlorophyll coordination is modelled in the density. Right, the same region from the X-ray study at 2.4 Å resolution^13^ lacks the density for the water, and the modeled Mg atom is not in the correct plane.

To confirm that the pre-PSI-1 is not a degradation product, we purified PSI from mature green leaves and determined its cryo-EM structure. The refined structure reached a resolution of 2.2 Å (Extended Data Fig. 1). We built the PSI model with a minimal clashscore of 4.6 (Table 1), which allowed us to produce correct chlorophyll coordination models with associated water molecules (Extended Data Fig. 2). Compared with previous models^13,14^, we identified a new gap chlorophyll CLA867 that is found between subunits PsaA and Lhca3 (Fig. 1a). CLA867 in our model is situated between CLA617 (3,011) of Lhca3 and CLA812 (1,109) of PsaA, within 10.6 Å and 13.3 Å, respectively (Fig. 1a). As the range is favourable for fast excitation energy transfer, our structure suggests that the CLA867 position rationalizes a previously undetected excitation energy path from LHCI to PSI. Importantly, CLA617 is situated between protein moieties with no direct coordination. Mature PSI from *A. sativa* shows a similar P700 oxidation profile compared with *Pisum sativum* (Fig. 2a), indicating a fully functional protein complex. The data are also consistent in terms of chlorophyll/P700 ratio (Fig. 2b).

**Extended Data Fig. 3:**
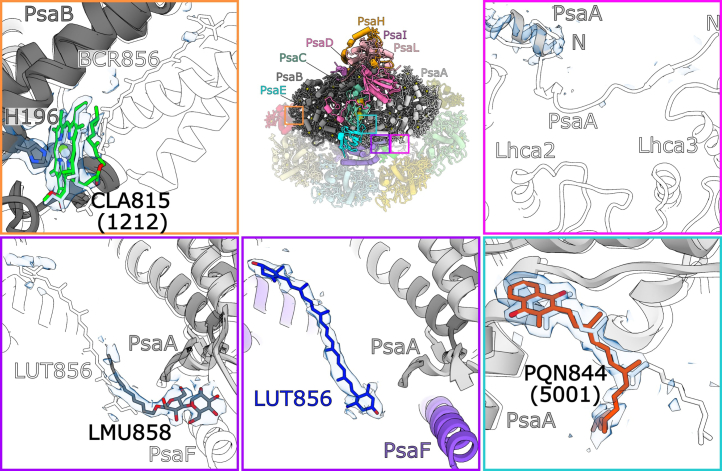
Superposition and comparison of pre-PSI-1 with mature PSI. The complete models are shown for pre-PSI-1 (coloured) and mature PSI (transparent). Structural alterations are shown in the close-up panels (mature PSI, white), and the coloured boxes indicate the position of the respective view in the structure.

Comparison between pre-PSI-1 and mature PSI revealed a series of structural alterations with respect to cofactor and protein conformation close to the interface between the PSI core and LHCI (Extended Data Fig. 3). First, the tail of chlorophyll CLA815 in pre-PSI-1 is ordered and occupies the space where the head group of β-carotene BCR856 resides in the mature structure. Second, the position of lutein 858 is partially preoccupied by a detergent molecule that probably represents a lipid as a structural holder in pre-PSI-1 until PsaF is incorporated into the complex. Third, phylloquinone PQN844 (PsaA) shows an altered conformation of the isoprenoid chain. Finally, the N terminus of PsaA is largely unstructured in pre-PSI-1 and forms a short two-turn helix (residues 31–40). In the mature PSI, this helix is replaced by a loop motif followed by an ordered N terminus stabilized mainly by interactions with Lhca3 (Extended Data Fig. 3).

**Extended Data Fig. 4:**
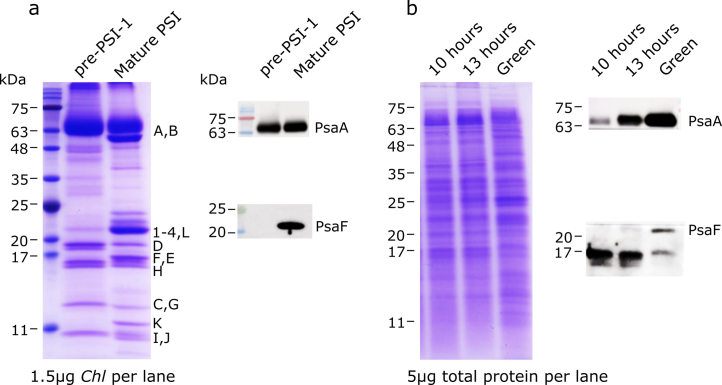
SDS-PAGE and immunoblotting analysis of PsaA and PsaF. **a**, PsaA and PsaF protein detection in purified pre-PSI and mature PSI preparations. Left, Coomassie stained SDS-PAGE. Right, corresponding immunoblots for PsaA and PsaF. **b**, PsaA and PsaF protein detection in thylakoid preparations from etiolated greening (10 and 13 hours of light) and normal green seedlings. Left, SDS-PAGE of the total protein isolated from thylakoid membranes. Right, Immunoblots showing the protein expression levels of PsaA and PsaF. The anti-PsaF anybody has weak affinity, and longer exposure results in a secondary band with lower molecular weight. The experiments were repeated independently with similar results three times. Source data

Unlike the previously reported assembly, mini-PSI^15^, the current intermediate pre-PSI-1 lacks the functionally critical subunit PsaF. To clarify a structural role for PsaF, we composed a protein–protein interaction map (Fig. 1c). This map shows that PsaF interacts with Lhca4 and PsaJ, which also interacts with Lhca2. In this way, PsaF is engaged in the binding of both antennae dimers: Lhca1–4 and Lhca2–3. Therefore, PsaF plays a key role in the association of LHCI, and in its absence in the pre-PSI-1 assembly intermediate the antenna proteins cannot stably bind due to the missing contacts (Fig. 1c). In *Arabidopsis*, PsaF-lacking PSI leads to distorted thylakoid grana, which gives rise to disorganization of the thylakoids^16^. We probed the presence of PsaF in the thylakoid membrane during greening and found no free PsaF (Extended Data Fig. 4). The missing PsaF and LHCI would affect the overall architecture of PSI, and thus potential inter-complex interactions in the thylakoid membrane. Overall, on the structural level, the assembly intermediate pre-PSI-1 suggests a link between PsaF, the light-harvesting antenna and thylakoid organization.

Next, we compared the kinetic properties of the assembly intermediate pre-PSI-1 with the mature PSI. Although the fully assembled PSI showed relatively high nicotinamide adenine dinucleotide phosphate (NADP) photoreduction activity of 633 ± 36 μmol NADPH (mg chlorophyll h)^−1^, the pre-PSI-1 showed no activity. We then inspected light-induced P700 photo-oxidation and P700^+^ reduction by Pc. This assay involved the use of different Pc concentrations, where a micromolar Pc concentration in the presence of ascorbate results in fully oxidized P700 upon illumination that is re-reduced in the dark, whereas excess Pc does not lead to P700 oxidation (Fig. 2c). An addition of NaCl slows down the electron transfer by weakening ionic interactions between Pc and PsaF, resulting in a light-dependent accumulation of oxidized P700 in the mature PSI. In contrast, for the pre-PSI-1, the electron transfers markedly slowed down (Fig. 2d), enabling accumulation of oxidized P700 independently of Pc concentration. Increasing NaCl has no effect on the electron transfer but rather on acceleration of P700^+^ re-reduction rates, which is attributable to the stronger hydrophobic interactions (salting out effect) between Pc and pre-PSI-1 in the absence of PsaF.

Overall, by using a natural system of organelle maturation, we visualized an intermediate in the biogenesis pathway of the plant PSI that contains less than half of its subunits; the intermediate was missing PsaF and all antenna proteins. Our study structurally characterized a native complex. In algal PSI, PsaH has been established as a regulatory subunit^2^, and a functional complex lacking PsaI and PsaL was reported^15^; however, these three subunits are already present in our non-catalytic assembly intermediate. Hence, stable binding of the conserved subunit PsaF is required for photoreduction and represents a critical step of the PSI biogenesis. Also in *Chlamydomonas* cells during a logarithmic growth phase, a subcomplex lacking PsaG, PsaK, LHCI and weakly bound PsaF has been identified, suggesting a universal mechanism^17^. However, in mutants deficient in PsaF, an assembled PSI could be observed^18^; thus additional transient intermediates may exist. Complemented by the feature of PsaF being inserted into the PSI from the thylakoid lumen^6^, structural data suggest that its attachment is mechanistically regulated. Therefore, PsaF represents a regulatory checkpoint that promotes the assembly and the consequent association of LHCI, effectively coupling it to function. These data are supported by analyses of PsaF-depleted PSI in *N. tabacum*^11^, and *Synechocystis* sp. PCC 6803 (ref. ^19^) showing that they form defined intermediates. As we do not observe unbound PsaF in the thylakoid membrane, the control might occur on the translational level, which was previously reported to play a key role during seed germination^20^ and chloroplast development^21^.

Collectively, our data confirm that PsaF is intimately linked to the photosynthetic functionality and assembly in a way that the pathway is dependent on the subunit accumulated in the thylakoid lumen during seedling greening. The exact mechanism of how PSI is modulated throughout the dynamic assembly to establish the catalytic complex remains to be explored, and our study opens the door for future work on more specific roles of other factors and their regulation. In addition, we define an accurate model of a plant PSI, including the complete set of pigments at their correct orientations, that will provide a reference plant PSI model for structural and molecular sciences.

## Methods

### Purification of PSI

Pre-PSI-1 was prepared from *A. sativa* (var. Saja 6) grown in the dark at 25 °C for 5 days. After the dark period, the plants were irradiated with cool-white fluorescent light at a photon flux density of 50 μmol photons m^−^^2^ s^−1^ for 10 h. Leaves (~470 g) were collected and ground in a blender with 900 ml of buffer containing 0.4 M sucrose, 30 mM tricine-NaOH (pH 8), 15 mM NaCl, 2 mM ascorbic acid, 1 mM PMSF and 1 μM pepstatin A. After filtration through cheesecloth, the suspension was centrifuged at 6,000*g* for 10 min, and again at 180,000*g* for 20 min. The pellet was resuspended in 200 ml buffer containing 10 mM tricine-NaOH (pH 8) and 150 mM NaCl and then pelleted through centrifugation at 180,000*g* for 20 min. The pellet was resuspended in 45 ml of buffer containing 10 mM tricine-NaOH (pH 8) and 0.4 M sucrose to a concentration of 1 mg ml^−1^ chlorophyll and solubilized with 1.5% *n*-dodecyl-β-d-maltoside (DDM). Following 30-min incubation on ice, the material was centrifuged at 176,000*g* for 20 min and applied on a diethylaminoethyl-cellulose column (2.5 cm × 13 cm) pre-equilibrated with 20 mM tris-tricine (pH 8.0) and 0.2% DDM. The material was eluted with 300 mM NaCl, concentrated 2× with 5% PEG-6000 precipitation and centrifuged through a sucrose gradient of 10–40% in an SW 40 rotor (Beckman) at 170,000*g* for 16 h. The green band containing PSI was collected, subjected to fast protein liquid chromatography chromatography, and then applied onto a 10–35% sucrose gradient and centrifuged at 336,000*g* for 4 h in an SW 60 rotor (Beckman). The green band was collected and sucrose was removed by buffer exchange.

Mature PSI was prepared from 7-day-old *A. sativa* (var. Saja 6) grown for 7 days in a 16:8 light/dark cycle at a photon flux density of 50 μmol photons m^−2^ s^−1^ and following a previously published protocol^8^.

### Kinetic measurements, P700 reduction and NADP^+^ photoreduction activity assay

Pc was codon optimized and heterologously expressed. The following experiments were performed generally as previously described^22^. P700 reduction was measured in a quartz cuvette containing 1 ml reaction mix (20 mM tricine-NaOH (pH 8), 5 mM MgCl_2_ and 0.05% DDM), 10 μmol ascorbate, 100 nmol methyl viologen, 16 μg chlorophyll PSI ml^−1^, and 50 pmol Pc of *Synechocystis* sp. PCC 6803 or 50 pmol cytochrome (Cyt) *c*_6_ (Cyt C533). P700 photo-oxidation and re-reduction by Pc and Cyt *c*_6_ were measured using a JTS-10 spectrophotometer by illuminating the sample with red light (705 nm) for 5 s. Changes in absorbance were measured by 2 ms of LED light flashes at 700 nm.

The NADP^+^ photoreduction activity assay was measured in a quartz cuvette. The 1 ml reaction mix (20 mM NaCl, 10 mM tricine-NaOH (pH 8), 0.5 mM MgCl_2_) was supplemented with 20 μmol ascorbate, 125 μg ferredoxin, 8.8 μg ferredoxin-NADP(+) oxidoreductase, 1 μmol NADP^+^ (Roche Diagnostics), 14 nmol Pc and PSI (14.4 μg chlorophyll). NADPH accumulation was measured at 340 nm (*ε* (the molar extinction coefficient) = 6,220 M^−^^1^ cm^−^^1^) using a Cary 60 spectrophotometer (Agilent Technologies) under continuous illumination with a 660-nm LED light (600 μE). The activity was calculated as μmol NADPH (mg chlorophyll h)^−1^.

### SDS–PAGE and immunoblotting

Isolated thylakoids and purified complexes were dissociated with SDS sample buffer. We used SDS–PAGE with a 17% gel to separate the proteins and then transferred the proteins to a nitrocellulose membrane using a wet transfer method (Bio-Rad Mini-PROTEAN Tetra Cell and Mini Trans-Blot Cell), according to the manufacturer’s instructions. The amount loaded corresponded to 1.5 µg of chlorophyll for purified complexes (Extended Data Fig. 4a) and 5 µg of total protein for thylakoids (Extended Data Fig. 4b). Protein concentration was measured with Bradford reagent (catalogue number 5000-0006; Bio-Rad), according to the manufacturer’s instructions. The antibodies used were anti-PsaA (AS06172; Agrisera) and anti-PsaF (AS011104; Agrisera).

### Cryo-EM data collection, processing and model building

Pre-PSI-1 (3 µl) and mature PSI at 2 mg ml^−1^ chlorophyll were applied on glow-discharged holey carbon grids and vitrified for cryo-EM structural determination using Leica EM GP (3-s blot at 20 °C and 100% humidity). Data were collected using EPU 1.9 software on a 300 kV Titan Krios G3 microscope (Thermo Fisher Scientific) equipped with a Gatan BioQuantum energy filter and a K3 Summit direct electron detector (Ametek). Videos were recorded using counting mode at a magnification of ×105,000, corresponding to a calibrated pixel size of 0.85 Å. A total of 3,324 micrographs at a total dose of 45 e Å^−2^ and 24,930 micrographs at 51 e Å^−2^ were collected for pre-PSI-1 and mature PSI, respectively, with a defocus range from −0.5 µm to −1.9 µm. Videos were imported into cryoSPARC 3.1 (ref. ^23^), and motion correction, contrast transfer function (CTF) estimation, picking and two-dimensional classification were performed on the fly during data collection using cryoSPARC Live (with blob picker and template picker). Ab initio models were generated with a subset of particles. Heterogenous and homogeneous refinement was performed for pre-PSI-1 and mature PSI, respectively. Particles (383,325 for mature PSI and 546,410 for pre-PSI-1) were converted into a STAR file format and imported into RELION 3.1.1 (ref. ^24^). Particles were re-extracted (unbinned) and processed in RELION using a box size of 400 pixels for pre-PS-1 and 500 pixels for mature PSI. For pre-PSI-1, three-dimensional (3D) classification with two classes was performed. One class with 169,213 particles of high-quality particles was selected and subjected to 3D refinement, which resulted in an overall resolution of 3.1 Å. CTF refinement, 3D refinement and Bayesian polishing followed by another round of CTF refinement were performed for pre-PSI-1 and mature PSI. Another 3D refinement resulted in an overall resolution of 2.1 Å for both the pre-PSI-1 and mature PSI. As the Lhca2–3 heterodimer in the mature PSI appeared to be loosely bound, we used focused classification (three classes) with signal subtraction with a mask around the Lhca2–3 region to improve the local density. One subclass showed a better-ordered Lhca2–3 region. The particles of this subclass (96,997) were selected, and the signal was reverted. A final 3D refinement of mature PSI resulted in an overall resolution of 2.2 Å with an improved density for the Lhca2–3 region.

Model building and real-space refinement of pre-PSI-1 were then carried out using Coot 9.1.4 (ref. ^25^). The completed model was then fitted into the mature PSI map, and the remaining protein chains were built using rigid-body-fitted PSI (PDB: 6YAC) as a starting model. All protein residues and pigments were fitted using Coot with locally optimized map weights. For the entire modelling in Coot^25^, restraint files for pigments and ligands were used that were generated using the Grade server (http://grade.globalphasing.org), as previously described^26^. Models were refined using Real-Space-Refine from the PHENIX suite^27^. The refinement protocol was optimized by adjusting for optimal refinement weight parameters. Iterations of validation, model building and refinement were carried out using MolProbity 4.2 (ref. ^28^), Coot 9.1.4 (ref. ^25^) and the PHENIX suite^27^. All the buried surfaces were calculated using the online tool PDBePISA v.1.52 (ref. ^12^).

### Reporting summary

Further information on research design is available in the Nature Portfolio Reporting Summary linked to this article.

### Supplementary information

Reporting Summary

### Source data

Source Data Extended Data Fig. 4 Unprocessed western blots and gels for right-hand side of each panel.

## Data Availability

Atomic coordinates and structure factors of pre-PSI-1 have been deposited in the Protein Data Bank under accession code 8BCW. Atomic coordinates and structure factors of mature PSI have been deposited in the Protein Data Bank under accession code 8BCV. The cryo-EM map of pre-PSI-1 has been deposited in the Electron Microscopy Data Bank under accession code EMD-15970. The cryo-EM map of mature PSI has been deposited in the Electron Microscopy Data Bank under accession code EMD-15969. Other atomic coordinates that were used in this study: 6YAC (https://www.rcsb.org/structure/6YAC; PSI-ferredoxin). Source data are provided with this paper.
