# Author Correction: Structure of plant photosystem I in a native assembly state defines PsaF as a regulatory checkpoint

**DOI:** 10.1038/s41477-024-01737-5

**Published:** 2024-06-10

**Authors:** Andreas Naschberger, Mariia Fadeeva, Daniel Klaiman, Anna Borovikova-Sheinker, Ido Caspy, Nathan Nelson, Alexey Amunts

**Affiliations:** 1 Science for Life Laboratory, Department of Biochemistry and Biophysics, Stockholm University, Solna, Sweden; 2 The George S. Wise Faculty of Life Sciences, Department of Biochemistry and Molecular Biology, Tel Aviv University, Tel Aviv, Israel; 3 Present Address: Westlake University, Hangzhou, China

**Keywords:** Cryoelectron microscopy, Photosystem I

Correction to: *Nature Plants* 10.1038/s41477-024-01699-8, published online 30 May 2024

In the version of the article initially published, Andreas Naschberger was incorrectly assigned two affiliations which has now been amended to a single affiliation (Science for Life Laboratory, Department of Biochemistry and Biophysics, Stockholm University, Solna, Sweden) in the HTML and PDF versions of the article.

