# Supplementary figures and images for: Structure of plant photosystem I in a native assembly state defines PsaF as a regulatory checkpoint
Source: Nat Plants. 2024 May 30;10(6):874–9. doi: 10.1038/s41477-024-01699-8 (PMC11208149; doi:10.1038/s41477-024-01699-8)

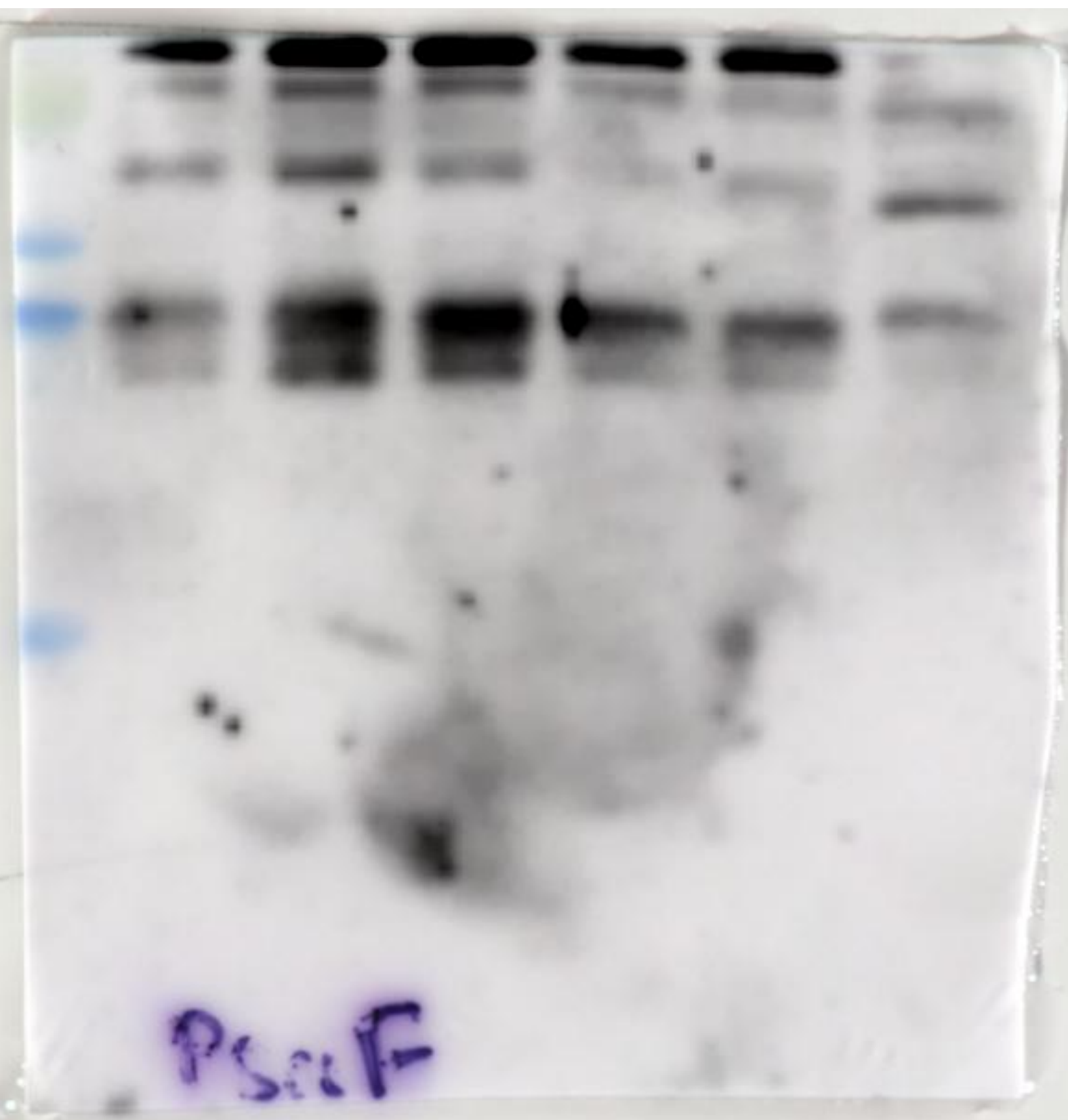

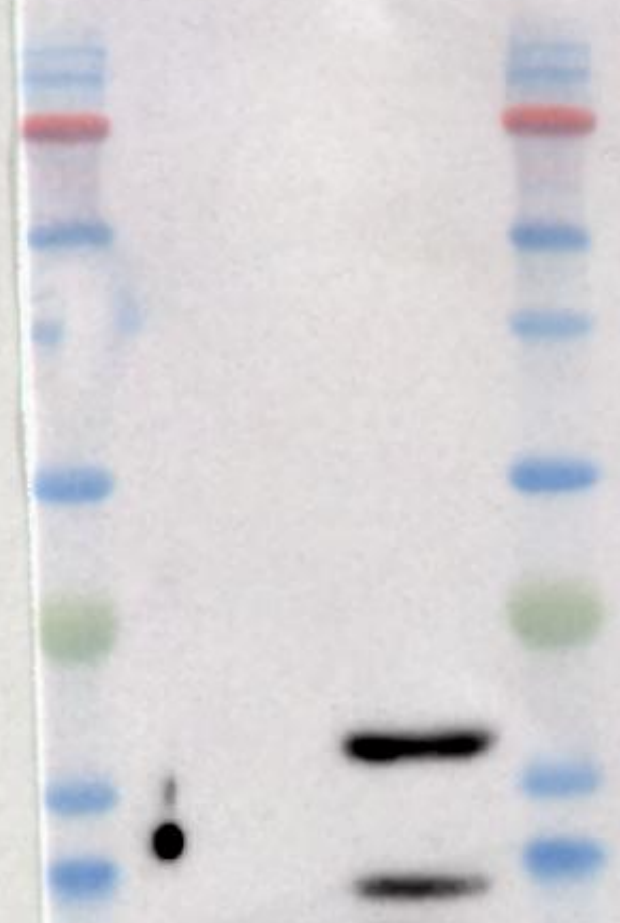

6-1

Psa F

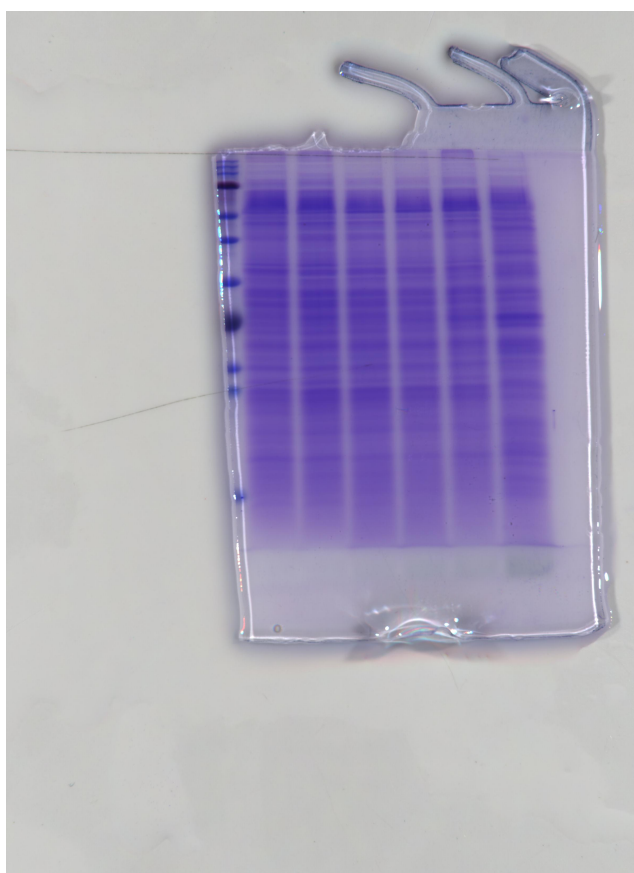

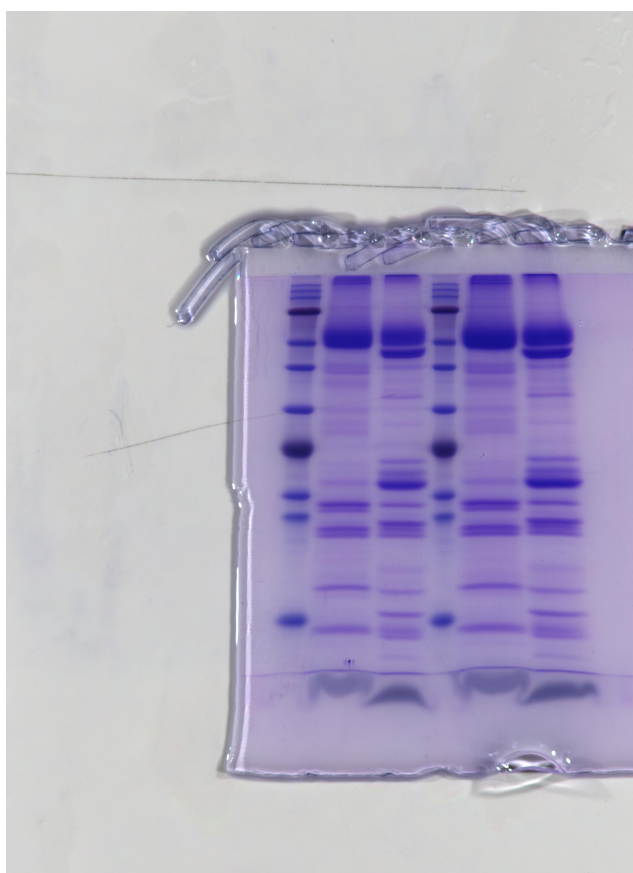

Supplement: Supplementary file 2 — Unprocessed western blots and gels for right-hand side of each panel. [file 41477_2024_1699_MOESM2_ESM.pdf]
